# Can patient gratitude expression boost innovative performance? The role of work meaningfulness and supervisory support

**DOI:** 10.3389/fpsyg.2022.1024211

**Published:** 2022-12-14

**Authors:** Bing Liu, Mengli Liu, Huijuan Wang, Yuanqi Yang, Ying Ma, Xin Wei

**Affiliations:** ^1^School of Management, Shandong University, Jinan, China; ^2^Zoina Land, Chengdu, China

**Keywords:** patient gratitude expression, nurses, innovative performance, work meaningfulness, supervisory support

## Abstract

Based on emotions as social information (EASI) theory, the current study proposed how and when patient gratitude expression could promote nurses’ innovative performance. Using a time-lagged data of 649 nurses from three class A tertiary hospitals in China, the results showed that patient gratitude expression was positively related to nurses’ innovative performance, and nurses’ work meaningfulness mediated such effect. Furthermore, supervisory support moderated the relationship of work meaningfulness with nurses’ innovative performance, as well as the indirect relationship between patient gratitude expression and innovative performance through work meaningfulness, such that the indirect relationship was stronger when supervisory support is higher. Our research helps to expand our understanding of how patient gratitude expression as an organizational external factor influences nurses’ innovation in healthcare, and meanwhile, provides management insights for hospital managers to focus on patient gratitude expression and enhance nurse innovation.

*“To nurses everywhere: You will interact with a patient for a moment but be in their minds forever. People will be forever grateful that you were their nurse.” Resha, 2018*

## 1 Introduction

The COVID-19 pandemic has challenged global health in the long term (Ward-Miller et al., 2021). As a primary component of front-line healthcare, nurses around the world have developed and tested innovative ways to deliver efficient, patient-centered care and achieve self-protection (Baron et al., 2021). For instance, a clinical nurse came up with the idea of putting small whiteboards inside the ward so that communication could take place without exposure, thus reducing the risk of infection among medical staff and also decreasing the amount of personal protective equipment used (Baron et al., 2021). Notably, in response to the challenges of global health, innovation has been a vital and necessary principle in nursing (Ward-Miller et al., 2021). Therefore, it is increasingly urgent to explore how to promote nurses’ innovative performance (i.e., the intentional development and implementation of novel and useful ideas within an organization in order to benefit role performance, a group, or an organization; Oldham and Cummings, 1996; Janssen and Van Yperen, 2004; Anderson et al., 2014) as the impact of the COVID-19 pandemic continues to expand.

Numerous studies have explored the antecedents of nurse innovation, including individual factors such as personality (Zappala et al., 2021) and psychological capital (Yan et al., 2020), task-related factors such as autonomy (Sonmez and Yildirim, 2019), and contextual factors like leadership and innovation climate (Bagheri and Akbari, 2018). Nevertheless, limited attention was given to the interpersonal triggers, such as patient-nurse relationship. Since interpersonal interactions can largely anchor both parties’ perceptions of work (Rosso et al., 2010), there is value, then, in uncovering the interpersonal cues for nurses’ innovative performance. In the specific context of patient-nurse relations, patient gratitude toward nurses has been considered one of the most valuable interactions in prior literature, and the importance of patient gratitude also has been highlighted (e.g., Converso et al., 2015; Starkey et al., 2019; Day, 2020). Accordingly, we intend to explore whether, how and when nurses’ perceived patient gratitude influences their innovative performance.

Drawing on emotions as social information (EASI) theory, we propose that perceiving patients’ grateful emotion can increase nurses’ innovative performance. That is, nurses may rely on cues from their patients to form views about their own ability to be creative (Shalley and Gilson, 2004). Specifically, EASI theory suggests that emotional expression in the workplace provides information to observers, triggering their affective reactions and inferential processes, which may in turn influence their cognition, attitudes, and behaviors (Van Kleef, 2009). As a positive emotional expression in the workplace, patient gratitude may induce nurses’ positive affective reactions as well as inferential processes, through which nurses realize why they are appreciated, thus developing their perceptions of the value of nursing work, namely, work meaningfulness (i.e., individual’s evaluation of one’s work of the extent to which the job is meaningful, valuable, and worthwhile; Hackman and Oldham, 1976). Given that nurses with high work meaningfulness possess a good sense of responsibility toward work (Steger et al., 2012), they are more motivated to think outside the box in order to better serve their patients. Meanwhile, when nurses encounter obstacles in the process of innovation, work meaningfulness empowers them with the determination to persevere (Lepisto and Pratt, 2017), thereby increasing the likelihood of implementation of ideas. Accordingly, we expect that patient gratitude expression will improve nurses’ innovative performance through enhancing nurses’ work meaningfulness.

**FIGURE 1:**
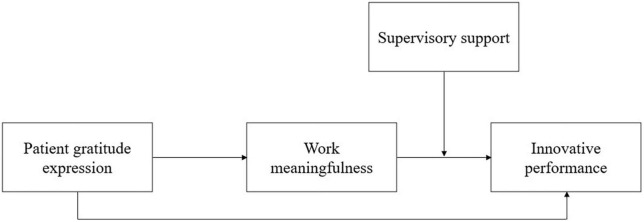
Research model.

Furthermore, since innovation is full of uncertainty and risk (Amabile, 1988), employees’ innovation requires external resources and support in addition to motivation, for instance, support from supervisors (Mishra et al., 2019), which has been tested as a key moderator for securing the implementation of innovative ideas (Cai et al., 2019). This is in line with the social interactionist approach in creativity and innovation research which suggests that personal and contextual characteristics jointly impact creativity and innovation (Scott and Bruce, 1994; Oldham and Cummings, 1996; Anderson et al., 2014). We thus posit that work meaningfulness interacts with supervisory support to affect nurses’ innovative performance such that when supervisory support is high as compared to low, they are more likely to make innovative achievements. Taken together, we propose a moderated mediation model, suggesting that patient gratitude expression has a positive indirect effect on nurses’ innovative performance *via* work meaningfulness, and supervisory support enhances this indirect effect (see Figure 1).

By examining these relationships, this study makes three contributions. First, by examining the effect of patient gratitude expression on nurse innovative performance, we extend the literature on nurse innovative performance. Existing research investigating the antecedents of nurse innovative performance has predominantly focused on personal characteristics and contextual factors (Shalley and Gilson, 2004; Anderson et al., 2014), ignoring the importance of interpersonal triggers, especially patient-nurse interactions. This manuscript draws attention to cues from interpersonal interactions and investigates the impact of nurses’ perceived patient gratitude on their innovative performance. Second, by relying on EASI theory and examining work meaningfulness as a mediator, this study reveals the theoretical mechanism through which patient gratitude expression affects nurses’ innovative performance. While previous research examining the mechanisms through which gratitude expression affects the recipient has primarily investigated the mediating roles of recipients’ cognition of the expresser and their relationship (Algoe et al., 2013; Williams and Bartlett, 2015), we introduce work meaningfulness as a mediator, which involves both affective and cognitive elements (Martela and Pessi, 2018), offering a new lens to understand how gratitude expression affects innovative performance. Finally, by investigating the moderating role of supervisory support, our study clarifies the boundary condition under which work meaningfulness affects nurses’ innovative performance. In doing so, we also enhance the understanding of how individual work-related cognition and organizational contextual factor jointly explain innovative performance (Cai et al., 2019).

## 2 Theoretical grounding and hypothesis development

### 2.1 Emotions as social information theory

Emotions as social information (EASI) theory indicates that emotions are not simple reflections of one’s internal affective states; they are also cues of social information, conveying important information to observers (e.g., the displayer’s personality or interpersonal intentions), and in turn, shapes observers’ consequent cognitions, attitudes, as well as behaviors (Van Kleef, 2009; Van Kleef et al., 2010). There are two distinct but mutually influential social-functional approaches of emotions: (1) affective reactions and (2) inferential processes (Van Kleef, 2009). Specifically, affective reactions refer to the emotions elicited in observers (Wang et al., 2017). Through immediate and primitive contagion, observers tend to catch the expresser’s emotions and develop reciprocal or complementary emotional states (Van Kleef et al., 2010). Yet, in the inferential pathway, specific emotions arise in response to appraisals and interpretations of specific situations, which provide a wealth of contextually relevant information about the expresser (Hareli and Rafaeli, 2008). Then, the observer makes a cognitive assessment of the expresser’s emotional performance and further determines their attitudes and behaviors (Van Kleef, 2009).

According to existing research, patients will express gratitude when they receive salvage and care from medical staff (Emmons and McCullough, 2003). Based on EASI theory, we posit that patient gratitude expression, as important information in the workplace for nurses, can trigger their affective reactions and inferences regarding patients’ intentions and aspirations, which in turn predict nurses’ attitudes and behaviors. Specifically, we predict that patient gratitude expression evokes nurses’ sense of work meaningfulness for nursing, making nurses more motivated and capable to engage in innovation.

### 2.2 Patient gratitude expression and nurses’ work meaningfulness

Work meaningfulness refers to the subjective experience and evaluation of how significant and intrinsically valuable people perceive their work to be (Rosso et al., 2010), which involves both affective and cognitive elements (Martela and Pessi, 2018). Employees may rely on social cues from others in the workplace to construct their own meaning of work (Wrzesniewski et al., 2003) and both emotional and cognitive processes are important in meaning-making (Park, 2010). Based on EASI theory, we propose that patient gratitude expression can offer interpersonal cues about their work and themselves (Wrzesniewski et al., 2003; Rosso et al., 2010), triggering nurses’ affective reactions and inferential processes, manifested in nurses’ work meaningfulness.

First, when perceiving a positive emotional experience of patients’ gratitude, nurses are immediately infected and feel positive emotions as well, which are closely related to work meaningfulness. In particular, King et al. (2006) pointed out that positive affective states are crucial in the experience of meaningfulness, no matter whether the positive affect is spontaneous or induced. Lee (2015) reviewed 28 studies for a concept analysis of “meaning in work,” suggesting that experiencing positive emotion at work is a critical attribute of “meaning in work.” Besides, Rosso et al. (2010) believed that the affective process strongly contributes to the positive relationship of interpersonal connectedness with work meaningfulness. Thus, patient gratitude expression can trigger nurses’ work meaningfulness through sending and eliciting positive emotional experience.

Second, patient gratitude expression evokes nurses’ inferential processes spontaneously and further enhances the nurses’ perception of the meaningfulness of their work. According to Rosso et al. (2010), there are four main pathways through which meaningful work is created or maintained—contribution, individuation, self-connection, and unification. Contribution is about the meaningfulness of actions perceived as significant or beyond one’s own benefits. Individuation is about how much one can experience self-efficacy and self-esteem through work. Self-connection reflects the meaningfulness of actions that enable individuals to realize and express themselves. Unification is about how much the work brings individuals into harmony with other beings or value systems.

In line with the above four pathways, we suggest that the inferential processes of patient gratitude expression can stimulate nurses’ work meaningfulness. In particular, the gratitude received by nurses may not only be a reward for their efforts in work but also a quality indicator for care representing the successful performance of the nursing job (Aparicio et al., 2019; Day, 2020). The recovery of the patient’s wellbeing depends on the nurses’ attentive care of the patient, verifying the contribution of nursing work (i.e., contribution pathway), and indicating nurses’ competence for this work (Blegen et al., 1992) (i.e., individuation pathway). This prediction is consistent with Grant and Gino’s (2010) arguments that gratitude expressions enhance helpers’ feelings of self-efficacy and social worth. In fact, in healthcare, expressions of gratitude, such as giving thanks, taking a bow, or literally a pat on the back, have been identified as meaningful recognition for health care professionals (Aparicio et al., 2019). In addition, patients’ acknowledgment of their work is consistent with their cognition of nursing, which aligns nurses more closely with their perceptions of themselves (i.e., self-connection pathway). Furthermore, nurses may infer a close relationship with the patients after perceiving patients’ grateful emotions, meeting nurses’ social needs in the workplace (i.e., unification pathway). We thus hypothesize:

Hypothesis 1. Patient gratitude expression is positively related to nurses’ work meaningfulness.

### 2.3 The mediating role of work meaningfulness

According to EASI theory, work meaningfulness, as a positive emotional experience and motivated state, can directly trigger individuals’ positive attitudes and behaviors. Existing research has demonstrated that work meaningfulness is positively associated with employees’ wellbeing (Arnold et al., 2007), job satisfaction (Ghislieri et al., 2019), and work engagement (Mostafa and El-Motalib, 2020). We further propose that work meaningfulness enhanced by patient gratitude expression can improve nurses’ innovative performance.

Perceiving work meaningfulness is a motivating state (Spreitzer, 1995), which is a key driver of individual creativity (Amabile, 1983; Atwater and Carmeli, 2009). Specifically, when employees feel the work is meaningful, they will show a strong intrinsic motivation (Hackman and Oldham, 1976) and a good sense of responsibility for work (Steger et al., 2012), thus being more motivated to engage in innovation (Oldham and Cummings, 1996). Meanwhile, Liang et al. (2021) suggested that people who experience their work as meaningful are more motivated to look beyond job routines and further stimulate their creativity. Additionally, given that innovation in the workplace is prone to failure due to its attributes of risk and uncertainty, work meaningfulness enhances nurses’ ability to resist setbacks in the pursuit of novel outcomes (Tu and Lu, 2013). Confronted by frustration and failure of innovative affairs, work meaningfulness empowers employees with a positive attitude and spirit to persevere (Lepisto and Pratt, 2017) and provides a compelling reason why nursing is worth doing. As such, the possibility of achieving success in innovation increases.

Emotions as social information theory indicates that emotional expressions in the workplace trigger affective reactions and inferential processes in observers, which in turn shape their behaviors (Van Kleef et al., 2010). The experience of work meaningfulness could be the most critical psychological state linking work context factors and work outcomes (Wang and Xu, 2019). Combined with Hypothesis 1, we further posit that work meaningfulness acts as a mediator in the relationship between patient gratitude and nurses’ innovative performance. Specifically, since patients are nurses’ service recipients, their emotional feedback can greatly influence nurses’ subjective feelings about their work and their innovation (Shalley and Gilson, 2004; Anderson et al., 2014). Through affective reactions and inferential processes toward patient gratitude, nurses view nursing as valuable and meaningful, thus having more motivation and the ability to achieve innovative performance. In sum, we hypothesize:

Hypothesis 2. Work meaningfulness mediates the relationship between patient gratitude expression and nurses’ innovative performance.

### 2.4 The moderating role of supervisory support

Thus far, we have proposed patient gratitude expression is positively related to nurses’ innovative performance through work meaningfulness. In this section, we further explored the boundary condition of this mechanism. That is, we answered the question of when patient gratitude expression will be more beneficial for nurses. The interactionist view on employee innovation suggests that individual factors and contextual factors interact to influence employees’ innovative process in the workplace (Oldham and Cummings, 1996). In addition, innovation in the workplace is generally characterized by high risk and uncertainty, while requiring a substantial investment of resources (Amabile, 1988; Scott and Bruce, 1994; Shalley and Gilson, 2004). In the same way, nurses may seek direct and explicit support from authoritative forces in their innovative work. Therefore, we posit that supervisory support interacts with work meaningfulness to impact their innovative performance.

As an interpersonal relationship, supervisory support reflects the beliefs employees hold concerning the extent to which supervisors provide work-related emotional and instrumental support (Mishra et al., 2019), which can strengthen the positive relationship between work meaningfulness and innovative performance in two ways. First, supervisory support can further protect and increase the motivation of innovation derived from work meaningfulness (Shalley et al., 2004). Specifically, supervisory support can provide nurses with crucial resources and information related to their work, including innovative work (Bacharach and Bamberger, 2007). It also helps nurses to establish a close connection with their supervisors and reduce the insecurity associated with the potential resistance and failure of innovation (Amabile, 1988), thus enhancing the willingness of nurses with a high sense of work meaningfulness to innovate. Besides, when perceiving supervisory support, those who innovate because of their presumption that their work is meaningful and valuable would have a sense of responsibility for resource utilization (Hoppe et al., 2017), thus stimulating their determination to create value for the organization (Hur et al., 2013). In this way, the willingness of nurses with a high sense of work meaningfulness to innovate is stimulated. By contrast, individuals who lack supervisory support may fear that innovation will bring failure and cost. Even though nurses have high work meaningfulness, they cannot reduce the uncertainty and interpersonal risk that they may encounter in undertaking creative activities, which thus undermines their motivation to innovate.

Second, in addition to psychological resources, supervisory support can also provide them with instrumental resources essential for innovation (Amabile et al., 2004). For instance, supervisors can provide nurses access to time, material resources, and people (Shalley and Gilson, 2004), thus guaranteeing the implementation of innovative ideas. In short, the innovative performance of nurses will be further improved with the dual effect of innovation motivation and innovation capacity endowed by supervisory support. Conversely, even though a high sense of work meaningfulness propels nurses to innovate, a lack of supervisory support would make it difficult to achieve results concerning innovation. Thus, we hypothesize:

Hypothesis 3. Supervisory support will moderate the positive relationship of work meaningfulness with nurses’ innovative performance, such that the relationship is stronger (vs. weaker) when supervisory support is higher (vs. lower).

Combining this rationale with the proposed indirect effect of patient gratitude expression on innovative performance *via* work meaningfulness, we further propose that supervisory support enhances this positive indirect effect. That is, patient gratitude expression stimulates nurses’ sense of meaningful work, which in turn drives them to engage in innovation. Furthermore, when obtaining emotional and instrumental support from supervisors, nurses’ motivation to innovate is strengthened with innovation implementation secured, demonstrating a stronger positive indirect effect between patient gratitude and innovative performance. We thus hypothesize:

Hypothesis 4. Supervisory support will moderate the positive indirect effect of patient gratitude expression on innovative performance *via* work meaningfulness, such that the indirect relationship is stronger (vs. weaker) when supervisory support is higher (vs. lower).

## 3 Methodology

### 3.1 Sample and data collection

We collected survey data from front-line nurses in three class A tertiary hospitals located in Jinan and Taiyuan, China. They have frequent contact with patients and can perceive patients’ gratitude. In particular, we first obtained the permission of the managing directors of each hospital to survey the nurses. Then, we coded the questionnaires confidentially and distributed them to the nurses with the help of the human resources departments of the three hospitals. To improve data quality, we informed each participant of the purpose of our study, their privacy, anonymity, and voluntary participation, and then offered 5–20 RMB as a reward for them.

To minimize common method variance, we followed Podsakoff et al. (2003) and collected three waves of data, each with a 2 month time lag. At time one, participants were asked to provide demographic information (e.g., age, gender, and working years) and complete measures of patient gratitude expression. At time two, nurses rated their sense of work meaningfulness. At time three, participants completed measures of innovative performance and supervisory support.

The survey was distributed to 1,200 nurses, with 1,067 responding at time one (for a response rate of 88.91%). Excluding the participants who left the hospitals, 1,053 participants were left for the second stage. At time two, 921 nurses responded (for a response rate of 76.75%) and were invited to complete the time three survey, in which 739 participated (for a response rate of 61.58%). After the three rounds of survey collection, we conducted a preliminary screening of the validity of the data. Firstly, we removed 34 samples with more than 20% missing values. Secondly, we added the attention test item in the questionnaires “To check whether you are attentive or not, please choose ‘strongly disagree’ for this question” and removed 43 samples that did not meet the response requirements; finally, considering the simple repetition and abnormal answering, we further removed 13 samples. Overall, with 90 excluded due to missing data or invalid information, 649 were retained for our analyses. Among the 649 nurses, 74.58% were female. They averaged 30.87 years of age (SD = 7.22), and had worked at their present organization for an average of 8.11 years (SD = 7.32).

### 3.2 Measures

To ensure the generalizability of scales, we adapted the well-established scales in previous research to measure our focal variables. We used Brislin’s (1970) translation-back translation procedure to translate the measures from English to Chinese. A seven-point Likert scale (1 = “strongly disagree,” 7 = “strongly agree”) was used.

#### 3.2.1 Patient gratitude expression

We adapted a three-item scale from Palmatier et al.’s (2009) gratitude items to the healthcare context. A sample item includes “Patients expressed their appreciation for my work.” The Cronbach’s alpha was 0.949.

#### 3.2.2 Work meaningfulness

We adapted a seven-item scale from Ashmos and Duchon (2000) to measure work meaningfulness. A sample item includes “The work I do is connected to what I think is important in life.” The Cronbach’s alpha was 0.959.

#### 3.2.3 Innovative performance

Nurses rated their innovative performance using a nine-item scale adapted from Janssen and Van Yperen (2004). A sample item includes “At work, I often create new ideas for improvements.” The Cronbach’s alpha was 0.953.

#### 3.2.4 Supervisory support

We adapted a four-item scale from Bacharach and Bamberger (2007) to measure supervisory support. A sample item includes “I can rely on my supervisor for advice or information when things get tough at work.” The Cronbach’s alpha was 0.965.

#### 3.2.5 Control variables

Previous research demonstrated that employees’ gender, age, and working tenure could be related to their innovative performance (Ng and Feldman, 2010). To rule out their potential confounding effects, we controlled for the effects of respondents’ gender, age, and working tenure. Gender was coded “0” for male and “1” for female, age was measured in years, and employee working tenure was coded in years.

## 4 Results

### 4.1 Descriptive statistics

Table 1 shows the results of means, standard deviations, and correlations of the variables in the study. As expected, patient gratitude expression was positively correlated with work meaningfulness (β = 0.429, *p* < 0.01). Patient gratitude expression and work meaningfulness were both significantly correlated with innovative performance (β = 0.388, *p* < 0.01; β = 0.410, *p* < 0.01), respectively. Work meaningfulness and innovative performance were both positively correlated with supervisory support (β = 0.540, *p* < 0.01; β = 0.365, *p* < 0.01), respectively. These findings provided initial support for our hypotheses.

**TABLE 1:** Descriptive statistics and correlations.

	*M*	SD	1	2	3	4	5	6
(1) Patient gratitude expression	5.125	1.103						
(2) Work meaningfulness	5.108	1.145	0.429**					
(3) Innovative performance	5.333	0.990	0.388**	0.410**				
(4) Supervisory support	5.061	1.232	0.351**	0.540**	0.365**			
(5) Gender	0.746	0.436	0.049	0.024	0.027	0.022		
(6) Age	30.874	7.219	−0.126**	−0.080*	–0.019	−0.139**	−0.160**	
(7) Working tenure	8.107	7.320	−0.098*	–0.051	0.001	−0.118**	–0.070	0.940**

*N* = 649. *p < 0.05; **p < 0.01.

### 4.2 Confirmatory factor analysis

Before testing our hypotheses, we conducted confirmatory factor analysis (CFA) in Mplus 7.4 to check the distinctiveness of our focal variables. To decrease the likelihood of identification problems in the CFA, we created parcels using the item-to-construct balance approach (Little et al., 2002) when constructs had more than six items as indicators. A baseline four-factor model and seven alternative models were developed. The result, as presented in Table 2, indicated that the four-factor (i.e., patient gratitude expression, work meaningfulness, innovative performance, and supervisory support) model showed a good fit to the data QQ (χ2 = 229.211, df = 59, CFI = 0.983, TLI = 0.978, RMSEA = 0.067, and SRMR = 0.023) and fit the data significantly better than all the three-factor models where any three of the four factors were combined. These findings provided support for construct distinction.

**TABLE 2:** Results of the confirmatory factor analysis (CFA).

Model	χ2	*df*	CFI	TLI	RMSEA	SRMR
Four-factor model	229.211**	59	0.983	0.978	0.067	0.023
Three-factor model 1: Combining PGE and WM	2422.159**	62	0.768	0.708	0.242	0.167
Three-factor model 2: Combining PGE and IP	2008.856**	62	0.809	0.759	0.220	0.146
Three-factor model 3: Combining PGE and SS	2110.063**	62	0.799	0.747	0.226	0.153
Three-factor model 4: Combining WM and IP	1965.277**	62	0.813	0.765	0.217	0.131
Three-factor model 5: Combining WM and SS	2194.108**	62	0.790	0.736	0.230	0.127
Three-factor model 6: Combining IP and SS	2054.550**	62	0.804	0.754	0.223	0.149
One-factor model: Combining all four factors	5848.838**	65	0.443	0.332	0.367	0.213

*p < 0.05; **p < 0.01. CFI, comparative fit index; TLI, Tucker–Lewis index; RMSEA, root-mean-square error of approximation; SRMR, standardized root-mean-square residual; PGE, patient gratitude expression; IP, innovative performance; WM, work meaningfulness; and SS, supervisory support.

### 4.3 Hypotheses testing

We used hierarchical multiple regression, combined with moderated mediation analysis in Mplus 7.4 to examine our hypotheses. Hypothesis 1 proposed that patient gratitude expression is positively related to nurses’ work meaningfulness. We tested Hypothesis 1 by regressing work meaningfulness on patient gratitude expression, supervisory support, and control variables (i.e., gender, age, and working tenure; Model 2). As shown in Table 3, the coefficient associating patient gratitude expression with work meaningfulness was statistically significant (β = 0.283, *p* < 0.01), supporting Hypothesis 1.

**TABLE 3:** Results of multiple hierarchical regressions.

Predictor	Model 1	Model 2		Model 3
	Innovative performance	Work meaningfulness	Innovative performance	Innovative performance
Intercept	2.817** (0.422)	1.908** (0.481)	2.458** (0.439)	4.125** (0.427)
Gender	0.022 (0.089)	−0.018 (0.085)	0.025 (0.086)	0.033 (0.086)
Age	−0.001 (0.015)	−0.016 (0.016)	0.002 (0.014)	0.002 (0.014)
Working tenure	0.010 (0.014)	0.019 (0.015)	0.006 (0.014)	0.006 (0.014)
Patient gratitude expression (PGE)	0.269** (0.039)	0.283** (0.045)	0.215** (0.040)	0.199** (0.041)
Supervisory support (SS)	0.215** (0.034)	0.414** (0.043)	0.137** (0.038)	0.140** (0.040)
Work meaningfulness (WM)			0.188** (0.044)	0.185** (0.046)
SS × WM				0.058* (0.024)
R^2^	0.214**	0.359**	0.245**	0.252**

*p < 0.05; **p < 0.01. Unstandardized coefficients are presented. Standard errors are reported in parentheses.

Hypothesis 2 predicted that work meaningfulness mediates the relationship between patient gratitude expression and nurses’ innovative performance. Results (see Model 2 in Table 3) showed that patient gratitude expression was positively related to work meaningfulness (β = 0.283, *p* < 0.01), and work meaningfulness in turn positively predicted innovative performance (β = 0.188, *p* < 0.01). In addition, bootstrapped indirect effects analysis (5,000 replications) indicated that the indirect effect of patient gratitude expression on innovative performance *via* work meaningfulness was significant (β = 0.053, 95% CI = [0.028, 0.085], see Table 4), supporting Hypothesis 2.

**TABLE 4:** Results of path analysis.

Path	Estimate	SE	95% CI	Hypothesis test
**Direct effects**				
Patient gratitude expression → Innovative performance	0.269**	0.039	[0.190, 0.342]	–
Patient gratitude expression → Work meaningfulness	0.283**	0.045	[0.195, 0.370]	Support H1
Work meaningfulness → Innovative performance	0.188**	0.044	[0.102, 0.275]	–
**Mediating effect**				
Patient gratitude expression → Work meaningfulness → Innovative performance	0.053**	0.014	[0.028, 0.085]	Support H2

*p < 0.05; **p < 0.01.

**FIGURE 2:**
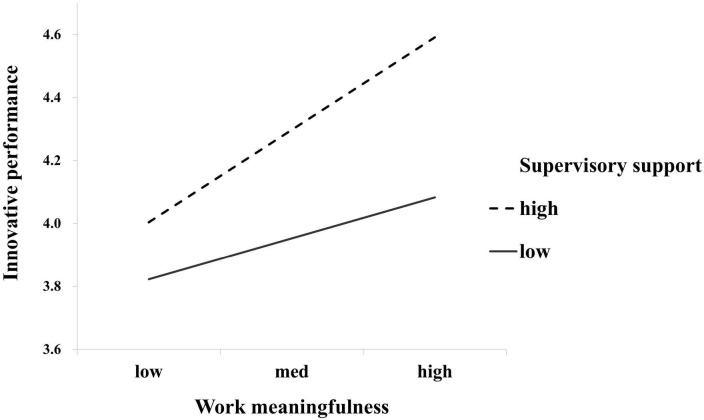
Interactive effect of work meaningfulness and supervisory support on innovative performance.

Hypothesis 3 predicted that supervisory support will strengthen the positive relationship of work meaningfulness with innovative performance. Based on Model 2, we added the interaction effect between work meaningfulness and supervisory support on innovative performance (Model 3). As shown in Table 3, the interaction effect was significant (β = 0.058, *p* < 0.05). Simple slope tests (see Table 5) showed that the relationship between work meaningfulness and innovative performance was stronger for nurses who perceived higher supervisory support (β = 0.256, *p* < 0.01) but weaker for nurses who perceived lower supervisory support (β = 0.114, *p* < 0.05). The difference in simple slope between the two conditions was 0.142 with a 95% CI of [0.021, 0.257]. The moderation effect is depicted in Figure 2. Thus, Hypothesis 3 was supported.

**TABLE 5:** Results of simple slope analyses and conditional indirect effects.

Supervisory support	Simple slope			Conditional indirect effects		
	Estimate	SE	95% CI	Estimate	SE	95% CI
High (+SD)	0.256**	0.054	[0.150, 0.364]	0.072**	0.017	[0.041, 0.109]
Low (+SD)	0.114*	0.055	[0.007, 0.225]	0.032*	0.017	[0.003, 0.071]
Difference	0.142*	0.060	[0.021, 0.257]	0.040*	0.017	[0.009, 0.078]

*p < 0.05; **p < 0.01.

We posited in Hypothesis 4 that the positive indirect effect patient gratitude expression has on nurses’ innovative performance *via* work meaningfulness is moderated by supervisory support, such that the indirect relationship is stronger (vs. weaker) when supervisory support is higher (vs. lower). The results are shown in Table 5. When supervisory support was higher, the positive indirect relationship was stronger (β = 0.072, 95% CI = [0.041, 0.109]) than when supervisory support was lower (β = 0.032, 95% CI = [0.003, 0.071]), and the difference between the two effects was significant (β = 0.040, 95% CI = [0.009, 0.078]), supporting Hypothesis 4.

## 5 Discussion

Since nursing staff innovation can propel an organization forward in improving patient satisfaction (Snide and Nailon, 2013), identifying ways of promoting nurses’ innovative performance is practically important. Drawing upon EASI theory, this study developed and tested a model addressing how and when patient gratitude expression is linked to nurses’ innovative performance. Findings from a multi-wave survey study suggested that patient gratitude expression can foster nurses’ innovative performance. In addition, when perceiving patients’ grateful emotions, nurses’ work meaningfulness would be enhanced, which in turn increases their innovative performance. Moreover, this study demonstrated that supervisory support would moderate the relationship between work meaningfulness and innovative performance, as well as the indirect effect of patient gratitude expression on innovative performance *via* work meaningfulness, such that these relationships would be stronger when supervisory support is higher.

### 5.1 Theoretical implications

The current study contributes to the literature in several ways. First, we extend the antecedents of nurses’ innovative performance, by shifting the perspective of improving nurse innovation from the inside to the outside of the organization and focusing on the interpersonal cues of nurses. Previous studies have explored personal characteristics (e.g., Yan et al., 2020; Zappala et al., 2021) and contextual factors within the organization as antecedents of nurses’ innovative performance, such as task contexts (e.g., Sonmez and Yildirim, 2019), leadership (e.g., Weng et al., 2015; Bagheri and Akbari, 2018) and organizational climate (Joseph, 2015), overlooking factor of interpersonal triggers outside of the organization. In fact, the literature on innovation suggested that future research should pay attention to actors outside of the organization (Anderson et al., 2014). Based on EASI theory, we contribute to the nurses’ innovative performance literature by studying patient gratitude expression as an important external predictor.

Second, we offer a new lens to understand the process through which the receipt of gratitude affects recipients’ innovative performance. Specifically, previous research has mainly drawn on attribution theory and find-remind-and-bind theory, and examined that recipients’ cognition of expresser and their relationship mediate the effect of gratitude on recipients’ outcomes (Algoe et al., 2013; Williams and Bartlett, 2015), which pay little attention on the affective nature of gratitude. Therefore, according to EASI theory, our study proposed and examined nurses’ work meaningfulness representing their affective reactions and inferential processes as a mediator in the relationship between patient gratitude expression and nurses’ innovative performance.

Third, by identifying supervisor support as the boundary condition of the relationship between work meaningfulness and nurses’ innovative performance, our study offers a more comprehensive understanding of when patient gratitude expression is more beneficial to nurse innovation. In particular, supervisory support can further increase the motivation of innovation derived from work meaningfulness and provide resources essential to innovation implementation (Shalley et al., 2004). In contrast, the implementation of nurse innovation will be hindered without supervisory support. Thus, by highlighting the complementarity between work meaningfulness and supervisory support as a facilitator of nurses’ innovative performance, we further enrich the literature on nurses’ innovative performance.

### 5.2 Practical implications

This study found that patient gratitude expression stimulates nurses’ work meaningfulness and thus increases their innovative performance. Hospitals and healthcare staff can encourage patients to express their gratitude and further increase the access to convey patients’ grateful expressions to the nurses (Day, 2020). For example, the medical secretaries of a general hospital in France systematically kept an archive of received thank you letters from the patients and further provided it to healthcare professionals (Herbland et al., 2017), which increased the mutual understanding between the two parties. However, hospitals should be careful not to regard gaining patients’ gratitude as the goal of their work and not to pursue this reward too much.

Second, research has shown that work meaningfulness is an important mechanism for the improvement of nurses’ innovative performance, and therefore nurses’ subjective perceptions of work meaningfulness should be enhanced. According to Rosso et al. (2010), organizations can create and maintain work meaningfulness in four pathways. Firstly, hospitals can accurately identify and appropriately use the strengths of employees, and further meet the individual needs of nurses to realize “individuation.” Secondly, hospitals should build a harmonious and inclusive working atmosphere to encourage the true expression of employees to achieve “self-connection.” Thirdly, as the sense of work meaningfulness is an intrinsic motivation for individuals, organizations and leaders should make nurses aware of the importance of nursing work to acknowledge their “contributions.” Finally, hospitals should regularly organize activities to strengthen the bonding of the nursing team to promote “unification.”

Third, the study found that a high level of supervisory support enhances the positive impacts of patient gratitude expression and work meaningfulness on innovative performance. Supervisors are therefore encouraged to provide adequate instrumental support, such as clarifying task roles and providing adequate job resources. Meanwhile, supervisors should also realize that relations support is equally important and further provide relevant support to nurses, such as being more closely involved with nurses, appreciating their creative ideas, and showing a high degree of trust in them (Cai et al., 2019).

### 5.3 Limitations and future research directions

Firstly, the respondents were from three class A tertiary hospitals in Jinan and Taiyuan, China. Although the sample size was rich and the reliability of the questionnaire was high, the geographical coverage of the sample sources was not extensive enough. Considering the differences in healthcare systems across countries (Bagheri and Akbari, 2018), we need to be cautious when generalizing our conclusions to other countries. Thus, we call for more studies including a large number of companies from different countries to enhance the generalizability of the findings in the future.

Secondly, we used a three-wave, time-lagged design to minimize common method bias, but we measured each construct only once and could not definitively establish causal inferences since the study is essentially cross-sectional. The results reported in the text can only represent the correlation between the variables and cannot accurately reflect the causal inference. Therefore, longitudinal studies or experimental investigations could be conducted in the future to better validate the causal inferences of the research model in this manuscript.

Thirdly, due to the specificity of nurses’ work, most of the innovation they generate are patient-oriented and cannot be accurately measured by leaders. In addition, the study is time-lagged and patients are highly fluid, so it’s also inappropriate to measure nurses’ innovation performance by patients. Therefore, we adopted the self-reported measures recommended by Madrid et al. (2014) to assess nurses’ innovative performance. Although existing studies suggested that the self-report of innovation was highly consistent with other-report (Madrid et al., 2014; Li et al., 2017), self-report innovation performance is still subjective in nature. We suggest that future studies can explore objective indicators to measure nurses’ innovation performance to increase the validity of the measurement.

Fourthly, the current study proposed and demonstrated that work meaningfulness and supervisory support as the mediator and the moderator in the relationship between patient gratitude expressions and nurses’ innovative performance. Yet, there may be other factors that could influence nurses’ innovative performance, such as nurses’ proactive personality. What is not well considered is that we focused primarily on nurses’ demographic information as control variables (Ng and Feldman, 2010), ignoring their personality traits. Hence, more control variables should be taken into account in the future.

Finally, this study only focused on the positive impact on nurses of gratitude from the people they serve. Yet, in the COVID-19 pandemic, healthcare workers around the world have made tremendous contributions, even, in many cases, sacrificing their lives. Collective and public expressions of gratitude to healthcare professionals have been springing up in mainstream media and social media around the world. In this context, the impact of collective gratitude and public gratitude on nurses can be expanded further in the future.

## Data availability statement

The original contributions presented in this study are included in the article/Supplementary material, further inquiries can be directed to the corresponding author.

## Ethics statement

The studies involving human participants were reviewed and approved by the School of Management, Shandong University. The patients/participants provided their written informed consent to participate in this study.

## Author contributions

BL and ML were involved in study conception and design the acquisition. BL, ML, and YM contributed to the data collection. YY and YM were involved in analysis and interpretation of the data. YY wrote the first draft of the manuscript. BL, ML, and XW were involved in critical revision of the manuscript. ML, HW, and YY were revised the manuscript according to all reviewers’ comments and completed the response letter. All authors reviewed the manuscript and have agreed on the final version.
